# Salinity induces dose-dependent metabolic reprogramming while maintaining apparent growth stability in *Limonium irtaense*

**DOI:** 10.1007/s00425-026-05042-7

**Published:** 2026-06-18

**Authors:** Diana M. Mircea, Carla Díaz-Tielas, Adela Sánchez-Moreiras, Pablo P. Ferrer-Gallego, Ricardo Mir, Jaime Prohens, Oscar Vicente, Monica Boscaiu, Sara González-Orenga

**Affiliations:** 1 Instituto Agroforestal del Mediterráneo (IAM), Universitat Politècnica de València, Camino de Vera s/n, 46022 Valencia, Spain; 2 Instituto de Conservación y Mejora de la Agrodiversidad Valenciana (COMAV), Universitat Politècnica de València, Camino de Vera s/n, 46022 Valencia, Spain; 3 Departamento de Bioloxía Vexetal e Ciencias do Solo, Facultade de Bioloxía, Universidade de Vigo, Campus Lagoas-Marcosende s/n, 36310 Vigo, Spain; 4 Instituto de Agroecoloxía e Alimentación (IAA), Universidade de Vigo, Campus Auga, 32004 Ourense, Spain; 5 Servicio de Vida Silvestre y Natura 2000, Generalitat Valenciana, Avda Comarques del País Valencia, 114, Quart de Poblet, 46930 Valencia, Spain; 6 Present Address: Swedish University of Agricultural Sciences, Southern Swedish Forest Research Centre, P.O. Box 190, 234 22 Lomma, Sweden

**Keywords:** Catecholamines, Endemic species, Halophyte physiology, Metabolomics, Osmolyte accumulation, Salt stress

## Abstract

**Main conclusion:**

***Limonium irtaense*** **maintains an apparently stable growth phenotype under salt stress through selective regulation of ion transport and extensive biochemical and metabolic reprogramming, revealing substantial stress adjustment costs.**

**Abstract:**

Although previous studies have examined some aspects of *Limonium irtaense*’*s* salt tolerance mechanisms, the metabolomic basis underlying its response to salinity remains unknown. Here, we provide the first comprehensive metabolomic characterization of salt stress responses in this species. Plants were exposed to increasing NaCl concentrations (0–0.3 M), and their responses were assessed through an integrated physiological, biochemical, and metabolomic approach, offering new insights into the mechanisms supporting adaptation to saline environments. Growth variables were not significantly affected by the salt treatments although the consistent downward trend in the mean values suggest a potential cost of salinity that may not be detectable at the salt concentrations applied. In contrast, biochemical and metabolomic analyses revealed pronounced internal adjustments, particularly at higher salinity. Proline, flavonoids, and polyols (xylitol, erythritol) accumulated progressively, whereas soluble sugars declined and lipid peroxidation increased, indicating enhanced oxidative stress. Untargeted metabolomics further revealed dose-dependent modulation of amino acid and carbohydrate pathways, alongside the novel induction of catecholamines, suggesting additional roles in antioxidant defense and ion regulation. These results highlight the tolerance of *L. irtaense*, supported by extensive biochemical and metabolic reprogramming. Beyond its conservation relevance, this study provides new insights into the adaptive strategies of halophytes and identifies metabolites that may represent candidate biochemical markers of salt tolerance. By integrating physiological and metabolomic evidence, this work advances understanding of how halophytes maintain growth under salinity while revealing the hidden physiological costs associated with stress tolerance.

**Supplementary Information:**

The online version contains supplementary material available at 10.1007/s00425-026-05042-7.

## Introduction

Salinity is one of the most widespread abiotic stresses, affecting plant distribution and functioning in coastal ecosystems. Excess salt imposes both osmotic and ionic constraints, reducing water uptake and causing ion toxicity, oxidative stress, and metabolic imbalance. Plants have evolved diverse strategies to tolerate saline environments, including ion compartmentalization, osmotic adjustment through compatible solutes, and reinforcement of antioxidant defenses (Munns and Tester 2008). Understanding these mechanisms is fundamental for predicting plant responses to increasing soil salinization and for identifying metabolic traits associated with stress resilience.

Halophytes, plants that are naturally tolerant to salinity, provide unique models to investigate the mechanisms of salt tolerance. Within them, the genus *Limonium* (Plumbaginaceae) represents one of the most diverse and physiologically specialized groups of coastal halophytes. Species of this genus exhibit remarkable tolerance to high NaCl concentrations that rely on a combination of structural, physiological and biochemical adaptations. These include specialized salt glands on the leaf surface that enable active secretion of toxic ions, such as sodium and chloride (Leng et al. 2018; Mi et al. 2021; Zhao et al. 2023), osmotic adjustment, ion homeostasis, and the accumulation of compatible solutes like proline and soluble sugars (Al Hassan et al. 2017). Under controlled conditions, several *Limonium* species tolerate salinity levels exceeding 0.8 M NaCl, often displaying significant changes in growth and metabolic activity (González-Orenga et al. 2019a, 2019b, 2020). Metabolomic studies have further revealed relevant stress tolerance mechanisms, including the accumulation of antioxidants and osmoprotectants that mitigate oxidative damage (Gagneul et al. 2007; González-Orenga et al. 2019b; Zhu et al. 2025), but comparative knowledge across species remains incomplete, and the roles of specific metabolic pathways remain largely unexplored.

*Limonium irtaense* P.P. Ferrer et al. is a narrow endemic species recently described by Ferrer-Gallego et al. (2015), found exclusively in the Serra d’Irta, northeastern Castelló, Spain. It is protected under regional legislation in the Valencian Community (Order 2/2022) due to its highly restricted distribution, small population size, and vulnerability to habitat disturbance. Despite its natural occurrence in saline habitats, only a few studies have addressed its physiological aspects (Mircea et al. 2023, 2026), and to date, no research has explored its metabolic responses under salt stress conditions. Understanding the tolerance mechanisms of this species is important not only for the conservation of this threatened endemism, but also for gaining insights into how halophytes adapt to fluctuating environmental conditions. This study investigates the mechanisms of salt tolerance in *L. irtaense*, with implications for understanding the stress responses of halophytes and for the conservation of narrowly distributed endemic species. While previous studies have addressed physiological and biochemical responses, this work places a particular focus on the untargeted metabolomic profiling of salt-induced changes in metabolite composition using GC–MS. In addition to growth and oxidative stress markers, we explore how increasing NaCl concentrations alter the plant’s metabolic pathways and osmolyte profiles. Through this integrated approach, we aim to uncover novel or underexplored metabolic mechanisms underlying salt tolerance in *L. irtaense*, with specific relevance to its conservation and ecological adaptation.

## Materials and methods

### Species under study

It has been reported that 27 *Limonium* species are present in the Valencian Community, Spain (Laguna et al. 2021). Surveys along the coastal cliffs in northern Castelló province have confirmed the presence of a rare and endangered endemic species, *L. perplexum* L. Sáez & Rosselló, co-occurring with *L. irtaense*.

*Limonium irtaense* is a perennial plant that grows 40–70 cm tall with a thick, woody caudex and, in general, with 1–3 floral stems. The leaves are basal, green at anthesis, and ovate–spathulate to orbicular–elliptic in shape, with visible lateral nerves and mucronate at the apex. The inflorescence is 10–30 cm long, with loose spikes that are 8–20 mm long, and flowers with violet, emarginated petals. Flowering occurs from June to September, whereas fruiting takes place between July and October. The chromosome number is 2*n* = 26. *Limonium irtaense* is closely related to the polyploid species *L. perplexum*, *L. virgatum*, *L. theanine*, and some members of the *L. delicatulum* complex. The new species should be traced back to either anagenetic events involving 2*n* = 26 ancestors or ancient hybridogenic processes (diploids with 2*n* = 16 and 2*n* = 18 are missing from the region where *L. irtaense* exists) (Ferrer-Gallego et al. 2015).

In the Valencian Community, *L. irtaense* is protected under the *Catálogo Valenciano de Especies de Flora Amenazadas* (Valencian Catalogue of Threatened Plant Species), according to the regional law (Orden 2/2022, dated February 16, issued by the Regional Ministry of Agriculture, Rural Development, Climate Emergency and Ecological Transition, updating the Valencian lists of protected plant and animal species).

### Plant growth and greenhouse treatments

Adult *L. irtaense* plants used in this study were obtained from the Centre for Forestry Research and Experimentation (CIEF) in València, Spain. These plants were grown from seeds provided by the Germplasm Bank of the Wildlife Service and the Natura 2000 network of the Generalitat Valenciana (accession code: 1391C114A), originally collected from Serra d’Irta, in Peñíscola, Castelló province. Plants were cultivated individually in 17 × 17 cm pots filled with a substrate composed of peat, perlite, and coconut fiber (4:1:1), which provided sufficient mineral nutrition throughout the experiment without additional fertilization. Stress treatments were initiated after three weeks of acclimation to the greenhouse, when plants were watered twice weekly with tap water. Five plants were assigned to each of the five treatments: control (0 M NaCl) and four salinity levels (0.05, 0.1, 0.2, and 0.3 M NaCl). The pH of the saline solutions was monitored at the time of preparation, with values of 7.86 (at 0 M), 7.86 (0.05 M), 7.86 (0.1 M), 7.84 (at 0.2 M), and 7.76 (at 0.3 M NaCl), ensuring that differences in nutrient bioavailability were minimized. For the first eight weeks, plants were irrigated twice a week with 0.3 L of the respective saline solution per pot. Over the following 11 weeks, irrigation frequency increased to three times per week, with 0.4 L applied per pot, two irrigations with the assigned NaCl solution and one with tap water across all treatments.

### Substrate electrical conductivity

The electrical conductivity (EC) of the substrate was initially monitored in each pot (*n* = 5) using a WET-2 sensor (Delta-T Devices, Cambridge, UK). However, as salinity increased over the course of the experiment, the EC values surpassed the measurement range of the sensor. Therefore, at the conclusion of the treatment period, substrate samples were collected and EC assessed in a 1:5 extract (EC_1:5_) using a Crison 522 conductivity meter (Crison Instruments SA, Barcelona, Spain). The EC_1:5_ values are expressed in mS cm⁻^1^.

### Morphological parameters

At harvest, the following traits were recorded: number of leaves, root length, the length of the longest leaf, and fresh weight (FW) of root and leaves. Root and leaf samples were oven-dried at 65 °C until a constant weight was reached, and the water content percentage (WC%) was calculated based on the difference between fresh and dry weights (DW). Biomass allocation was assessed as the Root/Leaf DW ratio and the leaf succulence (Delf's Index) according to the formula

$$Succulence=\frac{FW-DW}{LeafArea}$$

Fresh material was collected in parallel for biochemical and metabolomic analyses. Ion content measurements were performed using the corresponding dried material.

### Biochemical analysis

#### Photosynthetic pigments

Photosynthetic pigments were extracted from fully expanded, healthy leaves (excluding senescent material) collected at the end of the 19-week treatment period, to reflect the physiological status at harvest. Approximately 0.1 g of fresh leaf tissue was subjected to an extraction of photosynthetic pigments using 1 mL of ice-cold 80% (v/v) acetone. Centrifugation (13,300 g) at 4 °C and a 24 h dark incubation period came next. The supernatant was then diluted (1:10) with 80% acetone, and the absorbance was measured at 470, 646, and 663 nm. Following the Lichtenthaler and Wellburn (1983) equations, the concentrations of carotenoids (Carot), chlorophyll *a* (Chl *a*), and chlorophyll *b* (Chl *b*) were calculated and expressed as mg g⁻^1^ DW.

#### Osmolytes

Using approximately 0.1 g of fresh leaf tissue extracted in 0.5 mL of 3% (w/v) sulfosalicylic acid, the amount of proline (Pro) was measured in accordance with Bates et al. (1973). After reacting with 0.5 mL of acidified ninhydrin, the extract was incubated for one h at 95 °C. To extract Pro, 3 mL of toluene was added after cooling on ice. Proline concentration was determined using a standard curve and expressed as µmol g⁻^1^ DW after measuring the absorbance of the toluene phase at 520 nm.

The concentration of glycine betaine (GB) was calculated following Grieve and Grattan (1983), with certain adjustments (Valadez-Bustos et al. 2016). Freshly ground leaf tissue (0.15 g) was extracted in ultrapure water, agitated for 24 h at 4 °C, and centrifuged for 10 min at maximum speed (13,300 g). After mixing the supernatant with 2 N H₂SO₄ (1:1) and combining 125 µL of the mixture with 50 µL of cold KI–I₂, the reaction mixture was incubated for 16 h at 4 °C in the dark. Then, 1.4 mL of cold 1,2-dichloroethane was used to dissolve the pellet formed following 45 min of centrifugation at 0 °C. GB levels were calculated using a standard curve and expressed as µmol g⁻^1^ DW after the absorbance was measured at 365 nm.

The method by DuBois et al. (1956) was used to quantify total soluble sugars (TSS). 80% methanol extracts were combined with 5% phenol and concentrated sulfuric acid. After measuring the absorbance at 490 nm and calculating the TSS concentration using a glucose standard curve, the result was expressed in mg glucose equivalents (mg eq. gluc g⁻^1^ DW).

#### Oxidative stress markers

The Hodges et al. (1999) method was used to quantify the amount of malondialdehyde (MDA). 0.5% thiobarbituric acid (TBA) in 20% trichloroacetic acid (TCA) was added to methanol extracts, whereas controls just contained 20% TCA. Samples were chilled and centrifuged at 4 °C for 10 min at maximum speed (13,300 g) following a 15 min incubation period at 95 °C. The supernatant's absorbance was measured at 532 nm, and measurements at 600 and 440 nm were used to adjust for non-specific absorbance. The extinction coefficient ε₅₃₂ = 155 mM⁻^1^ cm⁻^1^ was used to calculate MDA concentrations following the formulae by Taulavuori et al. (2002).

Levels of hydrogen peroxide (H_2_O₂) were measured using Loreto and Velikova's (2001) methodology. After extracting approximately 0.05 g of fresh leaf tissue in 0.1% (w/v) trichloroacetic acid, samples were centrifuged at maximum speed (13,300 g) for 15 min at 4 °C. 500 µL of 10 mM potassium phosphate buffer (pH 7) and 1 mL of 1 M KI were mixed with the supernatant. H₂O₂ concentrations were calculated using a standard curve and represented as µmol g⁻^1^ DW after absorbance was measured at 390 nm.

#### Antioxidant compounds

The Blainski et al. (2013) method was used to assess total phenolic compounds (TPC). The Folin–Ciocalteu reagent was added to leaf methanolic extracts in the presence of sodium carbonate. The absorbance of the reaction mixtures was measured at 765 nm following a 90 min incubation period at room temperature in the dark. The standard curve was created using gallic acid (GA), and TPC concentrations were expressed as gallic acid equivalents (mg eq. GA g⁻^1^ DW).

Using the method by Zhishen et al. (1999), methanolic extracts were mixed with sodium nitrite and subsequently with aluminum chloride to determine the total flavonoid content (TF). The absorbance of the resulting solution was measured at 510 nm. TF concentrations were calculated and expressed as catechin equivalents (mg eq. C g⁻^1^ DW) using a catechin standard curve.

#### Ion content

The concentrations of the monovalent ions Na⁺, K⁺, Cl⁻, and the divalent Ca^2^⁺ were measured in roots and leaves according to Weimberg's (1987) and Cotlove’s (1963). About 0.1 g of ground dry material was extracted in boiling Milli-Q water, chilled on ice, and then centrifuged for 10 min at room temperature at 13,300 g. A Corning 410 Classic flame photometer (New York, USA) was used to measure the content of cations, and a Sherwood 926 chloride analyzer (Cambridge, UK) was used to measure the concentrations of Cl⁻.

#### Untargeted metabolomic analysis

Fresh leaves (0.1 g) were frozen and ground for metabolic assessment, followed by extraction, derivatization, and GC–MS analysis using the method by Lisec et al. (2006), with modifications from Misra et al. (2020). Briefly, enzymatic activity was stopped with cold pure methanol, ribitol was utilized as the internal standard, and chloroform was used as a solvent to extract polar-phase compounds. After drying in a vacuum centrifuge, each sample was derivatized with methoxyamine hydrochloride and *N*-methyl-*N*-trimethylsilyl-*N*-methyl trifluoroacetamide. Additionally, 10 µL of *n*-hydrocarbons mixture (alkane standard C10-C40, 50 mg/mL, Sigma Aldrich) was added for monitoring shifts in Retention Indices (RI).

GC–MS analysis employed an Agilent 7820 A gas chromatograph coupled with a 5975 C single quadrupole mass spectrometer and an RTX-5Sil MS capillary column (60 m × 0.25 mm × 0.25 μm) with the injector temperature set at 200 °C, and the source temperature maintained at 250 °C. Samples (1 μL) were injected with helium flow at 1 mL/min under the following programmed temperature: isothermal at 70 °C for 5 min, followed by a 5 °C/min ramp to 330 °C, and a final hold of 5 min. Electron impact (EI) mode at 70 eV was used to record the mass spectra, scanning at 50 − 600 m/z range, and using a 0.5 s scan period. Quality control samples and blank injections were included at regular intervals to monitor instrument performance and retention index stability. Peak intensities were used for the relative quantification of metabolites.

MS-DIAL software (ver.4.9.221218) was used to perform deconvolution, calibration, baseline filtering, peak extraction, alignment, identification height integration, and to normalize the data (Tsugawa et al. 2015). Peak identification was carried out by using 20 scans of peak width, and a minimum 1000 amplitudes of peak height A sigma window of 0.5 and EI-spectra cut-off of 5000 amplitudes were set for deconvolution. Retention time tolerance was set at 0.2 min for peak identification, m/z tolerance at 0.5 Da, EI similarity cut-off at 60%, and identification score cut-off at 80%. During the alignment parameter setting process, the retention time tolerance and the retention time factor were set to 0.5 min. Publicly available libraries were used for data annotation, such as the Golm Metabolome Database (Kopka et al. 2005), MassBank (Horai et al. 2010), and MoNA. Only metabolites that were putatively annotated and quantified were included. The identification of metabolites adhered to the guidelines set by the Metabolomics Standards Initiative (MSI) (Sumner et al. 2007)—Level 2 identification relied on matching spectra from a database with a match factor exceeding 80%. To further confirm these IDs, we manually checked the spectral fragmentation patterns for diagnostic ions and ensured that RI drift remained within acceptable limits for the entire batch. Still, because there could be isomeric overlaps or derivatization artifacts in complex catecholamine pathways, we consider these identifications as tentative.

#### Statistical analysis

The effects of the stress treatments on the attributes examined were assessed using one-way analysis of variance (ANOVA). Tukey’s post hoc test (*P* < 0.05) was used to evaluate group differences after the null hypothesis was rejected. SPSS Statistics software (IBM SPSS Statistics) was used to analyze the data. SRplot was used to graph and visualize Principal Component Analysis (PCA) and cluster the experiment's 25 plants on the 29 morphological and biochemical variables (Tang et al. 2023). This study utilized MetaboAnalyst 6.0 for data processing, statistical analysis, and visualization of metabolomics data. The data were normalized, log-transformed, and standardized using Pareto scaling. Unsupervised principal component analysis (PCA) was used to create a score plot for group discrimination and a loading plot to identify metabolites contributing to this separation. Univariate analysis was performed using one-way analysis of variance (ANOVA) and post hoc test (*P* ≤ 0.05) to identify significant metabolites across treatments. Student's t tests (*P* ≤ 0.05) were used to compare salinity treatments with the control group. Pairwise comparisons between salinity levels and control were performed to explore dose-dependent metabolic responses and detect significant differences among salt treatments. Hierarchical clustering heatmaps were generated to illustrate patterns of variation. A pattern hunter analysis was performed to identify metabolites whose accumulation patterns were positively or negatively correlated with increasing salinity. A pathway analysis was performed using MetPA to detect the affected pathways after each NaCl treatment using *Arabidopsis thaliana* as the reference organism database.

## Results

### Substrate analysis and growth parameters

After 19 weeks of treatment, the electrical conductivity (EC) of the substrate was measured (1:5 extract) and is presented in Table 1. The control group exhibited the lowest EC (3.6 mS cm^−1^), while EC values increased progressively with higher NaCl concentrations. The 0.3 M NaCl treatment resulted in the highest EC (32.5 mS cm⁻^1^), indicating a substantial rise in ionic concentration in the substrate due to the elevated salinity.

**Table 1:** Electrical conductivity upon completing the treatment period, EC values (1:5)

Treatment (NaCl)	0 M	0.05 M	0.1 M	0.2 M	0.3 M	Average SE
EC_1:5_ mS cm^−1^	3.6 a	17.2 b	26.3 bc	29.8 bc	32.5 c	2.4

Values shown are the means (*n* = 5), and the average SE (standard error). For each parameter, the different lowercase letters indicate significant differences between treatments for each determined variable, according to the Tukey’s post hoc test (*P* < 0.05)

The effects of increasing salinity on various growth parameters are summarized in Table 2. Root length was not significantly affected by the salt treatments. In contrast, leaf number showed a significant reduction from the lowest salinity level (0.05 M NaCl) onwards, decreasing from approximately 100 in control plants to 61 at 0.05 M NaCl. No further significant differences were observed at higher salinity levels, indicating that leaf number remained stable despite increasing salinity.

**Table 2:** Effect of salt stress on the growth parameters of *L. irtaense* after 19 weeks of NaCl treatments

Treatment (NaCl)	0 M	0.05 M	0.1 M	0.2 M	0.3 M	Average SE
RL (cm)	23.1 a	23.8 a	24.7 a	28.3 a	28.0 a	2.4
LLL (cm)	16.20 a	14.20 a	16.80 a	15.60 a	15.30 a	0.99
Nr_Leaves	99.8 b	61.0 a	58.4 a	60.2 a	70.0 a	5.3
FW_Roots (g)	15.1 a	12.5 a	10.0 a	8.7 a	10.6 a	1.8
FW_Leaves (g)	57.5 a	37.9 a	43.7 a	40.2 a	52.9 a	6.6
DW_Roots (g)	6.8 a	5.4 a	4.4 a	3.5 a	4.2 a	0.8
DW_Leaves (g)	12.2 a	8.9 a	9.9 a	8.7 a	11.3 a	1.6
Root/Leaf DW	0.6 a	0.7 a	0.5 a	0.4 a	0.4 a	0.1
Leaf succulence (mg/cm^2^)	26.1 a	30.3 a	36.0 a	31.9 a	40.7 a	4.9
WC_Roots (%)	56.4 a	56.9 a	56.4 a	59.7 a	60.6 a	2.0
WC_Leaves (%)	76.9 a	74.9 a	77.0 a	77.9 a	78.6 a	1.4

Values shown are the means (*n* = 5), and the average SE (standard error). For each parameter, the different lowercase letters indicate significant differences between treatments for each determined variable, according to the Tukey’s post hoc test (*P* < 0.05)

*RL* root length, *LLL* longest leaf length, *Nr_Leaves* number of leaves, *FW* fresh weight, *DW* dry weight, *WC* water content

Root fresh and dry weights showed a decreasing trend with increasing NaCl concentrations. Control plants had the highest root biomass (15.1 g FW), while plants treated with 0.2 M NaCl recorded the lowest (8.7 g FW). Although the observed differences were not statistically significant, the consistent decrease in the average values of root biomass may reflect a negative trend associated with increasing salinity.

A similar pattern was observed for leaf fresh and dry weights, as well as the root-to-leaf dry weight ratio. Control plants had the highest leaf biomass (57.5 g FW), whereas those subjected to the 0.2 M NaCl treatment had the lowest (40.2 g FW). As observed for roots, slight reductions in leaf biomass were observed across all salt treatments, supporting the negative trend in growth parameters associated with increasing salinity, even though the differences were again not statistically significant. Leaf succulence increased with salinity, as expected in halophytes like *L. irtaense*, where higher soil salinity typically leads to more succulent leaves to help dilute the salt. However, no statistically significant differences were found across treatments.

Water content (WC%) in roots also remained relatively stable across treatments, ranging from 56.4% (control) to 60.6% (0.3 M NaCl). Similarly, only a minor variation was observed in leaves, with WC% values between 74.9% (0.05 M) and 78.6% (0.3 M), indicating that leaf hydration and turgor were not significantly affected by salinity.

### Biochemical parameters

The biochemical responses of *L. irtaense* plants to salt treatments are summarized in Table 3. Chlorophyll *a* (Chl *a*), chlorophyll *b* (Chl *b*), and carotenoids (Carot) concentrations did not differ significantly between treatments although average levels were higher in the presence of 0.2 M NaCl for all three pigments.

**Table 3:** Effect of stress treatments in the leaf content of photosynthetic pigments, osmolytes, oxidative stress biomarkers, and antioxidant compounds after 19 weeks of salt treatments

Treatment (NaCl)	0 M	0.05 M	0.1 M	0.2 M	0.3 M	Average SE
Chl *a* (mg g^−1^ DW)	0.54 a	0.44 a	0.54 a	0.68 a	0.50 a	0.22
Chl *b* (mg g^−1^ DW)	0.36 a	0.37 a	0.28 a	0.71 a	0.56 a	0.16
Carot (mg g^−1^ DW)	0.48 a	0.42 a	0.42 a	0.72 a	0.56 a	0.15
Pro (µmol g^−1^ DW)	1.7 a	3.6 a	18.6 b	29.3 c	49.5 d	1.1
GB (µmol g^−1^ DW)	2.24 ab	1.19 ab	0.88 a	1.73 ab	2.55 b	0.37
TSS (mg eq. gluc g^−1^ DW)	36.1 b	52.7 c	42.0 bc	29.8 b	15.0 a	2.8
MDA (nmol g^−1^ DW)	180.1 a	258.8 b	275.1 b	279.2 b	289.8 b	15.5
H_2_O_2_ (µmol g^−1^ DW)	0.96 a	1.14 a	0.49 a	0.57 a	0.76 a	0.2
TPC (mg eq. GA g^−1^ DW)	38.7 ab	73.5 c	77.9 c	47.4 b	34.3 a	2.7
TF (mg eq. C g^−1^ DW)	3.94 a	8.08 b	9.29 bc	11.85 c	11.23 c	0.64

*Chl a and Chl b* chlorophylls *a* and *b*, *Carot* carotenoids, *Pro* proline, *GB* glycine betaine, *TSS* total soluble sugars, *MDA* malondialdehyde, *H*_*2*_*O*_*2*_ hydrogen peroxide, *TPC* total phenolic compounds, *TF* total flavonoids, *gluc* glucose, *GA* gallic acid, *C* catechin

Values shown are the means (*n* = 5), and the average SE (standard error). Different lowercase letters indicate significant differences between treatments for each determined variable, according to the Tukey’s test (*P* < 0.05)

Proline (Pro) content increased significantly with salinity, following a clear dose-dependent pattern. While no significant differences were detected at 0.05 M NaCl compared to the control (1.7 µmol g⁻^1^ DW), a significant increase was observed at 0.1 M. Pro levels continued to rise with increasing salinity, reaching a maximum of 49.5 µmol g⁻^1^ DW at 0.3 M NaCl, about 30-fold higher than in the non-stressed control, supporting the Pro role as a stress-responsive osmoprotectant. Glycine betaine (GB) content was variable and relatively low for all salt treatments, differing significantly from the control group (2.24 µmol g⁻^1^ DW) only at 0.3 M NaCl (2.55 µmol g⁻^1^ DW). Total soluble sugars (TSS) exhibited a non-linear trend in response to salinity. The highest concentration was observed at 0.05 M NaCl (52.7 mg eq. gluc g⁻^1^ DW), which was significantly higher than the control, whereas its lowest value (15.0 mg eq. gluc g⁻^1^ DW), significantly lower than the control, was measured at the highest salt concentration tested, 0.3 M NaCl (Table 3). Therefore, while TSS do not appear to contribute consistently to osmotic adjustment, the strong accumulation of Pro suggests a major role in osmotic balance under high salinity conditions. MDA, a lipid peroxidation and membrane damage marker, increased significantly under all NaCl treatments, reflecting oxidative stress induced by salinity. Control plants showed the lowest MDA level (180.1 nmol g⁻^1^ DW), whereas all salt-treated plants had higher average MDA contents, increasing in a concentration-dependent manner from 260 to 290 g nmol g⁻^1^ DW, approximately, although without statistically significant differences across the NaCl treatments. Concentrations of hydrogen peroxide (H_2_O_2_) were relatively constant in all salt treatments, with no significant differences observed among treatments (Table 3).

Total phenolic compound (TPC) content was significantly affected by the NaCl treatments. The highest values were observed at the lowest salinities, 0.05 M and 0.1 M NaCl, both approximately twofold higher than the control and significantly different from it. TPC concentration decreased at higher salinities, reaching a value not significantly different from the control in the presence of 0.3 M NaCl. Total flavonoid content (TF) followed a distinct trend, showing a significant increase with rising salinity, with the highest values recorded at 0.2 M and 0.3 M NaCl, 11.85 and 11.23 mg eq. C g⁻^1^ DW, respectively, about threefold higher than the control, non-stressed plants (Table 3).

### Ion contents in roots and leaves

**Fig. 1:**
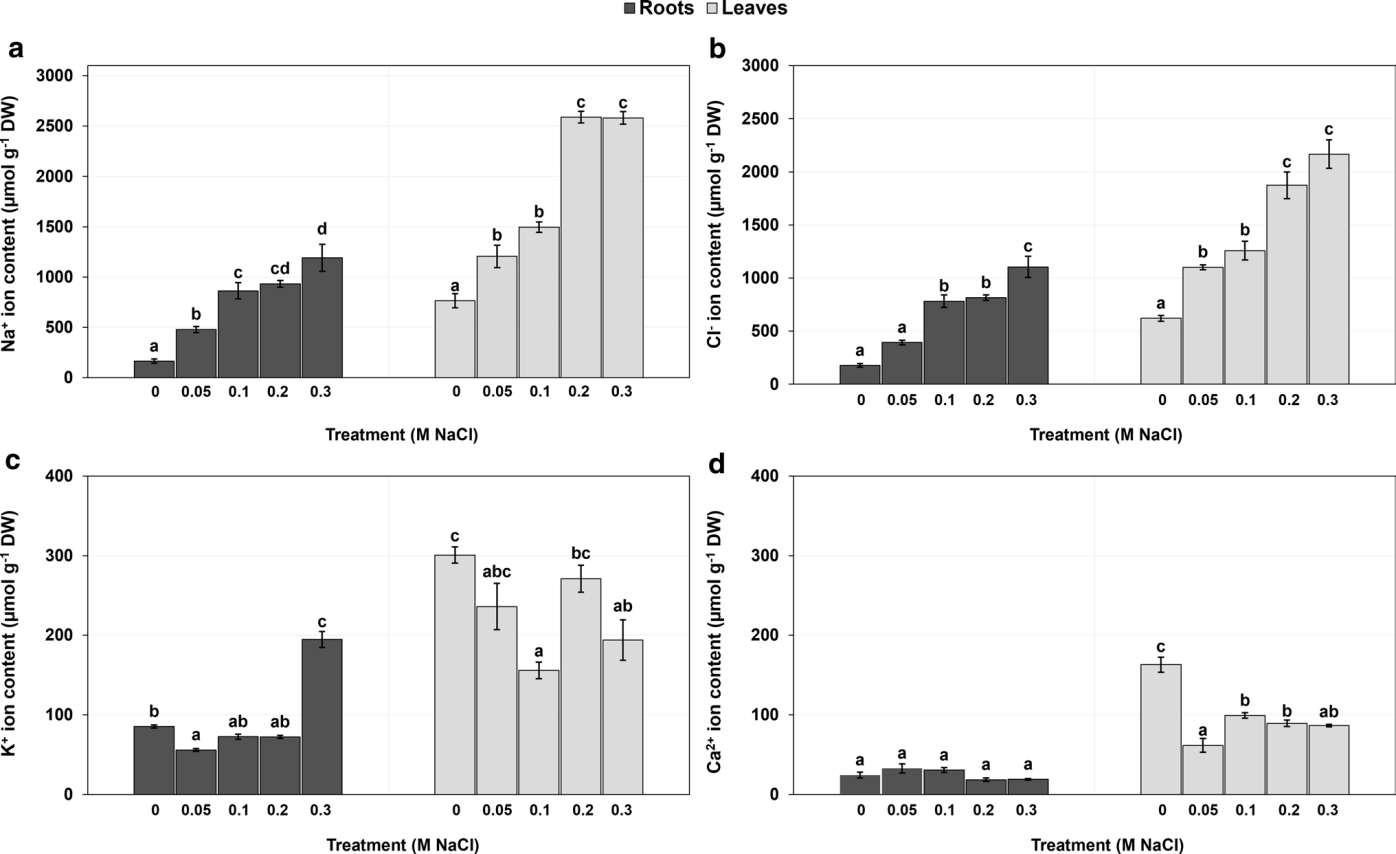
Effect of salt stress treatments on the content of ions in roots and leaves. **A** Sodium. **B** Chloride. **C** Potassium. **D** Calcium. Values shown are means ± SE (*n* = 5). The same lowercase letters indicate homogeneous groups between treatments for root and for leaves, respectively, according to the Tukey’s test (*P* < 0.05)

A significant increase in sodium (Na⁺) content in leaves and roots was detected in response to increasing NaCl concentrations (Fig. 1A). At 0 M NaCl (control), Na⁺ content was negligible in both tissues. With increasing salinity, Na⁺ accumulated progressively and significantly in both roots and leaves, displaying a dose-dependent pattern. Notably, Na^+^ concentrations were consistently higher in leaves than in roots at all salinity levels, which implies active translocation of the cation from roots to aerial parts. At the highest salinity (0.3 M NaCl), Na⁺ content reached 2,600 µmol g⁻^1^ DW in leaves, compared to ~ 1,200 µmol g⁻^1^ DW in roots, representing over a twofold difference (Fig. 1A). Similarly, chloride (Cl⁻) content increased significantly in response to NaCl treatments in both plant organs and was significantly higher in leaves than in roots across all treatments. At the highest salinity (0.3 M NaCl), Cl⁻ content in leaves reached nearly 2,200 µmol g⁻^1^ DW, double than that accumulated in roots (Fig. 1B). Changes in potassium (K⁺) concentrations (Fig. 1C) showed a different pattern to that of Na⁺ and Cl⁻. In roots, K⁺ contents remained relatively stable across most treatments, with a significant increase only in the presence of 0.3 M NaCl. In leaves, average K⁺ levels showed a general decreasing trend under salt stress when compared to the control, for which the highest concentration was recorded, even though the differences were not always statistically significant. Nevertheless, for each salinity treatment, K^+^ contents were higher in leaves than in roots (Fig. 1C). Calcium (Ca^2^⁺) content showed different patterns in roots and leaves. In roots, Ca^2^⁺ levels were low and did not change significantly at any tested salt concentration. However, leaf Ca^2^⁺ contents in control plants were much higher than in roots and decreased significantly in response to all salt treatments (Fig. 1D).

### Principal component analysis (PCA)

**Fig. 2:**
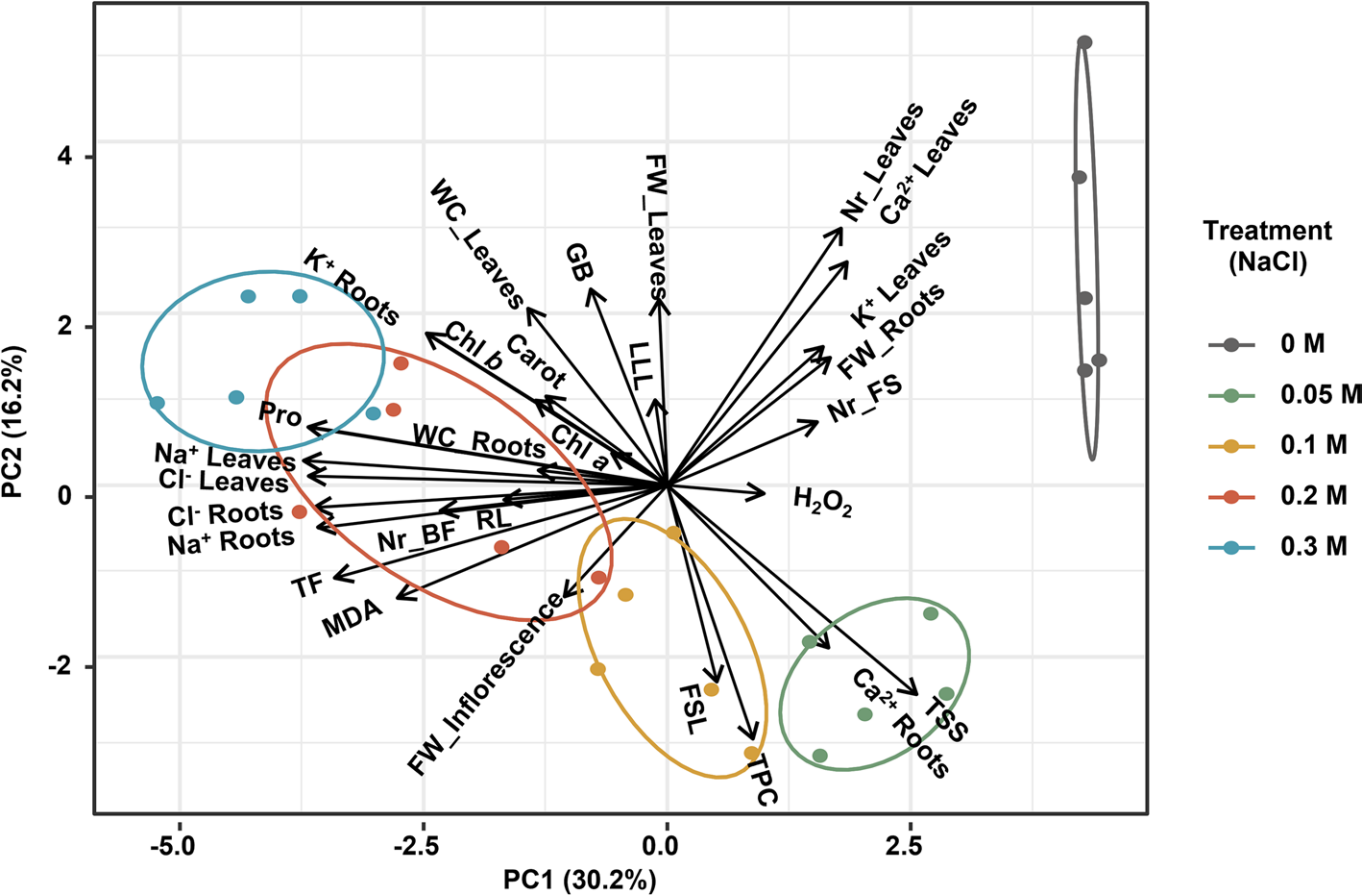
Principal component analysis biplot of growth and biochemical data of *Limonium irtaense*. *RL* root length, *LLL* longest leaf length, *Nr_Leaves* number of leaves, *FW* fresh weight, *DW* dry weight, *WC* water content, *Chl a and Chl b* chlorophylls *a* and *b*, *Carot* carotenoids, *Pro* proline, *GB* glycine betaine, *TSS* total soluble sugars, *MDA* malondialdehyde, *H*_*2*_*O*_*2*_ hydrogen peroxide, *TPC* total phenolic compounds, *TF* total flavonoids

The relationship among morphological, biochemical, and ion accumulation parameters in response to increasing NaCl concentrations is reflected in the PCA biplot (Fig. 2). The treatments were separated along the two principal components (PC1 and PC2), which together accounted for 47.4% of the total variance. PC1, explaining 30.5% of the total variance, showed a clear separation between salt-treated and control plants (0 M), where increased NaCl concentrations are associated with more negative PC1 values. Control plants cluster together on the positive side of the PC1, strongly associated with traits, such as number of leaves (Nr_Leaves), K⁺ and Ca^2+^ in leaves, and root fresh and dry weight (FW_Roots, DW_Roots). In contrast, plants subjected to higher salinity (0.2 M and 0.3 M) shifted toward the negative side of PC1. This cluster was characterized by the significant accumulation of toxic ions (Na⁺ and Cl⁻) in both roots and leaves, and the activation of osmotic and antioxidant defense mechanisms, specifically Pro, TF, and MDA. Notably, leaf succulence and root length (RL) were also positioned on this negative axis, indicating their strong association with highly saline environments.

Separation along PC2, which accounts for 16.9% of the total variance, suggests additional biochemical differentiation among treatments. Moderate salinity levels (0.05 M and 0.1 M NaCl) were distinctly separated along this axis, clustering in the lower region of the plot. Notably, root K⁺ content was positioned in the upper region of PC2, whereas traits associated with root Ca^2^⁺ content, total phenolic compounds (TPC), total soluble sugars (TSS), and higher root/shoot DW ratios (roots/leaves) were located on the negative side of PC2. This distribution suggests their stronger association with lower salinity levels. In contrast, variables, such as root fresh weight (FW_Roots), number of leaves (Nr_Leaves), and K⁺ and Ca^2^⁺ contents in leaves, contributed more to the positive side of PC2, aligning with the overall performance of control (0 M NaCl) plants.

While K⁺ root content showed an upward trend at the highest salinity level tested (0.3 M), the photosynthetic pigments (Chl *a*, Chl *b*, and Carot) and GB were situated closer to the center of the plot, reflecting their relative stability across the tested salinity gradient under the applied experimental conditions.

### Metabolomic analysis

#### Global effects of salinity on the metabolome

**Fig. 3:**
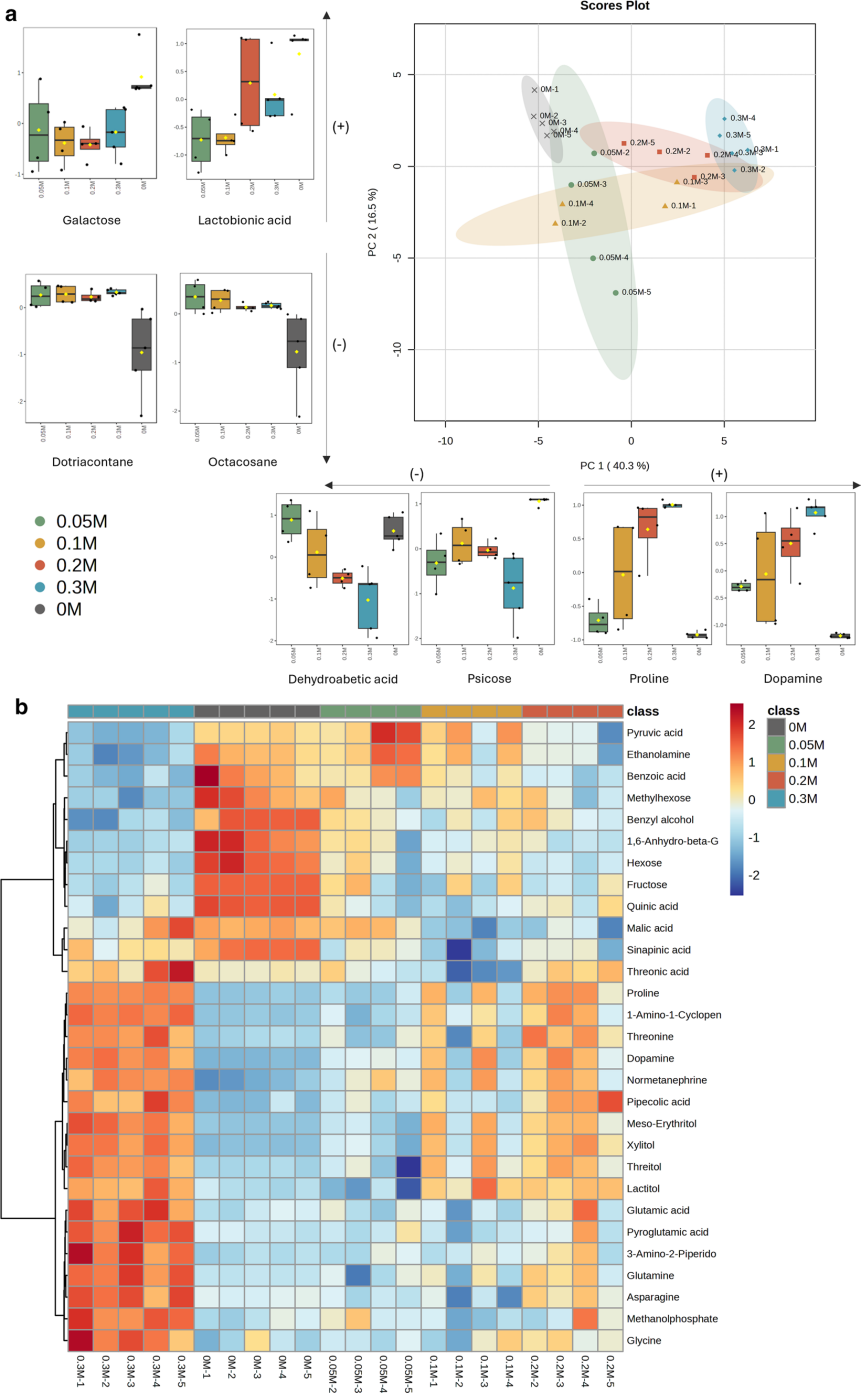
**A** Unsupervised principal component analysis (PCA) scores plot of all the annotated metabolites data from *Limonium irtaense* under different NaCl concentrations and boxplots of the top two metabolites with the highest positive and negative loadings for PC1 and PC2. **B** Heatmap and hierarchical clustering of metabolites showing the relative up-accumulation (red) or down-accumulation (blue) of the very highly significant (*P* < 0.001) metabolites altered after one-way ANOVA (each row of the heatmap corresponds to a metabolite) in the control and the four treatments (each column corresponds to a replica). 0 M NaCl, gray color; 0.05 M, green color; 0.1 M, mustard color; 0.2 M, orange color; 0.3 M, blue color

A total of 133 metabolites, mostly involved in primary metabolism, were annotated in the leaf tissues of *L. irtaense*. Unsupervised PCA based on these metabolites revealed a clear grouping of samples according to the NaCl treatments (Fig. 3A). The first two principal components (PC1 and PC2) accounted for 56.8% of the total variance, with PC1 explaining 40.3% and PC2 explaining 16.5%.

PC1 distinctly separated the samples based on increasing salinity, with control plants (0 M NaCl) clustering on the negative side and those treated with 0.3 M NaCl on the positive side. Samples from intermediate salinity treatments were distributed progressively along PC1, indicating a dose-dependent shift in metabolic profiles. This component thus captured the gradual metabolic changes associated with increasing salt stress. PC2 further contributed to differentiation, primarily separating the control group from all salt-treated samples.

According to the PCA loadings plot (Suppl. Table S1), PC1 was mainly influenced by metabolites, such as dopamine, proline, and tyramine, all showing strong positive loadings exceeding 0.20. In contrast, a range of sugars, including glucose, fructose, sucrose, and sugar alcohols, such as iditol, were negatively correlated with PC1, suggesting a general decline in carbohydrate levels in response to salinity (Suppl. Table S2). PC2 was primarily driven by lactobionic acid, which had a strong positive loading value (0.238), highlighting its importance in distinguishing control from salt-treated samples.

One-way ANOVA revealed that 87 out of the 133 annotated metabolites were significantly affected by the NaCl treatments (*P* < 0.05) (Suppl. Table S3). Among these, 50 metabolites showed stronger significance (*P* < 0.01), and 29 were significant at a highly stringent threshold (*P* < 0.001). These 29 compounds were visualized in a heatmap (Fig. 3B), providing an overview of their trends across the treatments and showing their relative accumulation patterns. The top ten metabolites with the highest F values in the ANOVA analysis (Suppl. Table S3) were hexose, 1-amino-1-cyclopentanecarboxylic acid, lactilol, 3-amino-2-piperidone, quinic acid, pyroglutamic acid, xylitol, meso-erythritol, benzyl alcohol and asparagine. These compounds exhibited distinct accumulation patterns. Hexose, quinic acid and benzyl alcohol accumulated significantly more in control conditions than in salt-treated ones. In contrast, the remaining compounds showed increased accumulation under salt treatments, with varying trends. Specifically, 1-amino-1-cyclopentanecarboxylic acid, xylitol, meso-erythritol, and lactilol displayed a clear dose-dependent increase in response to salinity. However, for lactilol, this increase was not significant at the lowest salinity level. Lastly, asparagine, 3-amino-2-piperidone, and pyroglutamic acid showed significant accumulation only at the highest salt concentration, suggesting a threshold-dependent metabolic response.

### Pairwise comparisons: control vs. individual salt concentrations

**Fig. 4:**
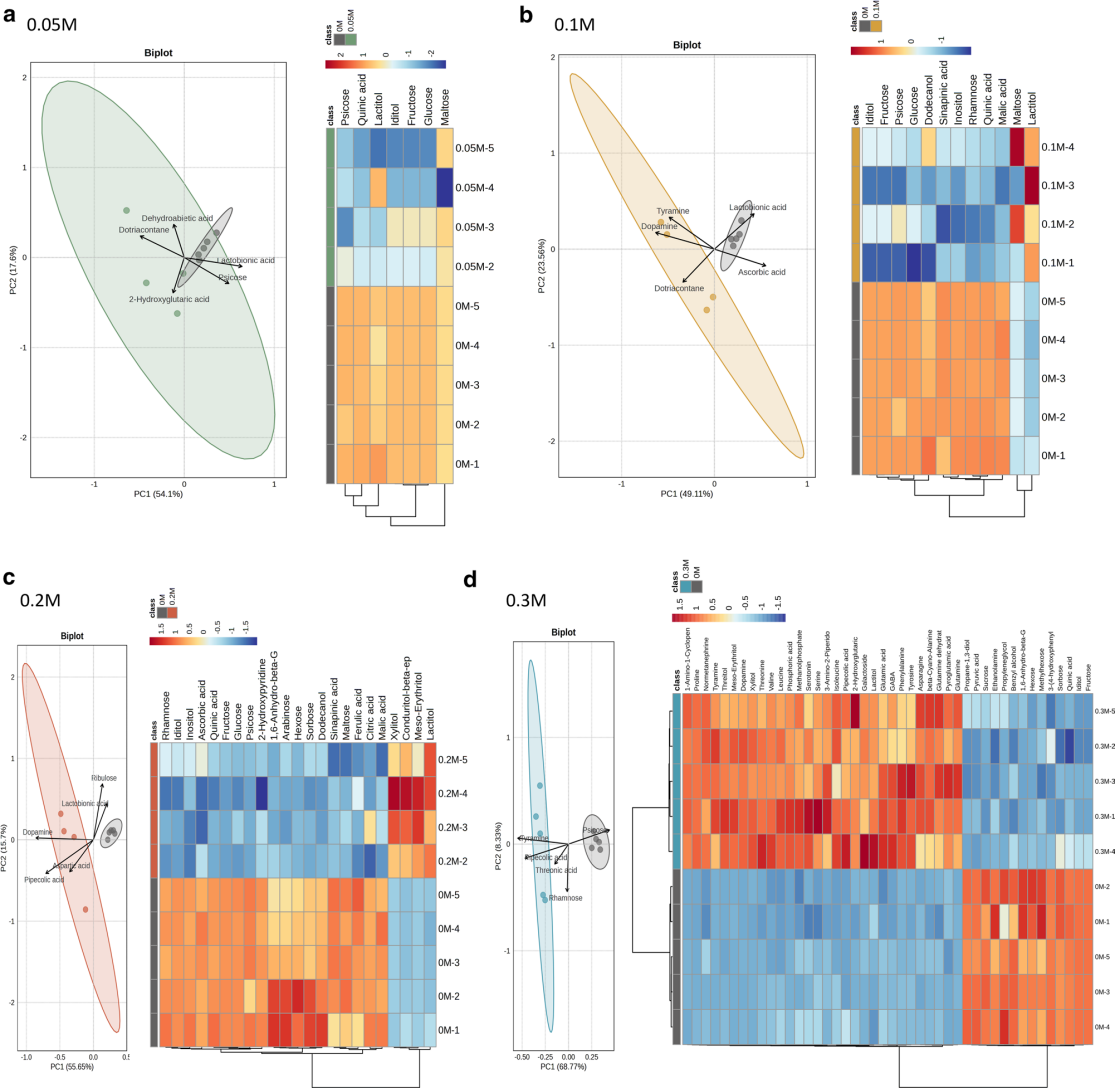
Biplot displaying the percentages of variance explained by the principal components of the PCA, along with the most important metabolites contributing to the separations and overlay heatmaps of the significantly (*P* < 0.001) altered metabolites after t test showing the relative up-accumulation (red) or down-accumulation (blue), each row of the heatmap corresponds to a metabolite, and each column corresponds to a replica. **A** 0.05 M, **B** 0.1 M, **C** 0.2 M, **D** 0.3 M NaCl. Complete data shown in Suppl. Table S4, S5, S6 and S7

A t test was conducted to compare the control with each of the salt treatments (0.05, 0.1, 0.2, and 0.3 M NaCl), and statistical significance was determined using False Discovery Rate (FDR)-adjusted *p* values. The comparison between the control and the 0.05 M NaCl treatment revealed that low salinity significantly affected 20 out of 133 identified metabolites (FDR < 0.05). Among these, only four (dopamine, gentisic acid, normetanephrine, and tyramine) were up-accumulated under low salt conditions. The remaining 16 metabolites were more abundant in the control, with lyxose, quinic acid, ascorbic acid, and sinapic acid being the most prominent (Suppl. Table S4, Fig. 4). At 0.1 M NaCl, 41 metabolites exhibited significant differences compared to the control (Suppl. Table S5). Of these, 12 were up-accumulated, including lactitol, threitol, and xylitol, which showed the most pronounced increases. Notably, dopamine and normetanephrine also remained elevated under moderate salinity. The pairwise comparison between the control and the 0.2 M NaCl identified 57 significantly altered metabolites (Suppl. Table S6), with 29 up-accumulated and 23 down-accumulated. Several metabolites that responded at lower concentrations, including dopamine, normetanephrine, and ascorbic acid, continued to show increased accumulation. Additionally, proline was strongly up-accumulated. Finally, at the highest salinity level (0.3 M NaCl), 88 metabolites were significantly altered (Suppl. Table S7), with 44 displaying highly significant differences (*P* < 0.001). Of the 88 metabolites affected, 41 showed higher accumulation in plants subjected to severe salt stress, highlighting proline, 1-amino-1-cyclopentanecarboxylic acid, dopamine, xylitol, valine and meso-erythritol as the most strongly up-accumulated. In contrast metabolites, such as sucrose, fructose, and iditol, exhibited markedly lower levels under salt stress compared to the control.

To further investigate metabolite responses, a pattern hunter analysis was performed using a linear correlation model. This approach identified several metabolites with strong positive correlations to salt concentration, suggesting a dose-dependent accumulation. Proline showed the highest correlation (*r* = 0.999), followed by 1-amino-1-cyclopentanecarboxylic acid, xylitol, dopamine, and valine, all with correlation coefficients above 0.98 (Suppl. Table S7).

Pairwise comparisons among the salt treatments revealed that the metabolic response was highly dependent on the intensity of the salt stress. No significant differences were observed between 0.05 M and 0.1 M NaCl, between 0.1 M and 0.2 M, or between 0.2 M and 0.3 M. However, when comparing 0.05 M with 0.2 M NaCl, glycerol was the only metabolite showing a significant change, with a notable decrease under moderate salt stress (Suppl. Table S8). A more pronounced shift was detected when comparing 0.05 M and 0.3 M NaCl, with 47 metabolites significantly altered, of which 37 were up-accumulated in response to high salinity (Suppl. Table S9). Among the most strongly up-accumulated were proline, dopamine, 1-amino-1-cyclopentanecarboxylic acid, meso-erythritol, and asparagine, all showing *p* values below 0.0001. Similarly, the comparison between 0.1 M and 0.3 M revealed 26 significantly different metabolites, including 18 that increased under severe salt stress (Suppl. Table S10). The most significant changes were observed for pyroglutamic acid, methanol phosphate, xylonic acid, phosphoric acid, and methylhexose, with notably increases in their levels in response to high salinity. Interestingly, no significant differences were detected between 0.2 M and 0.3 M NaCl concentrations.

### Pathway enrichment analysis of salt-induced metabolic changes

All salinity levels (0.05, 0.1, 0.2, and 0.3 M NaCl) were subjected to pathway enrichment studies in comparison to the control (0 M). Supplementary Tables S11–S14 present the significantly enriched pathways at each concentration.

Based on a false discovery rate (FDR) threshold of less than 0.05, only two metabolic pathways were substantially enriched at the lowest salinity level (0.05 M NaCl) in comparison to the control (Suppl. Table S11). These were glutathione metabolism and betalain biosynthesis. The glutathione metabolism pathway showed moderate pathway impact (0.14) and was significantly enriched (FDR = 0.0052), suggesting an early activation of antioxidant defense mechanisms in response to low salt stress. In contrast, betalain biosynthesis, although statistically significant (FDR = 0.0015), presented no pathway impact (impact = 0), indicating limited topological relevance in the dataset.

At 0.1 M NaCl, pathway enrichment analysis revealed a clear intensification of the metabolic response compared to the lowest salt level, with 13 metabolic pathways significantly enriched (FDR < 0.05) (Suppl. Table S12). The most significantly affected was inositol phosphate metabolism (FDR = 1.10 × 10⁻⁶), although showing modest topological impact (0.09). Another prominently enriched pathway was the citrate cycle (TCA cycle) (FDR = 9.41 × 10⁻^5^; impact = 0.19). Additionally, pathways related to carbohydrate metabolism, such as galactose metabolism (FDR = 0.0013; impact = 0.68), glyoxylate and dicarboxylate metabolism (FDR = 0.0013; impact = 0.29), and glycerolipid metabolism (FDR = 0.0013; impact = 0.16), were also significantly enriched. Several other pathways with high biological relevance were enriched as well, including starch and sucrose metabolism, pyruvate metabolism, and ascorbate and aldarate metabolism. The phenylpropanoid biosynthesis pathway (FDR = 0.0050) also appeared significantly enriched.

At 0.2 M NaCl, the metabolic response of *L. irtaense* became considerably more complex, with a total of 49 significantly (FDR < 0.05) enriched pathways (Suppl. Table S13). Among the most enriched pathways were those related to primary metabolism, such as galactose metabolism (FDR = 0.00060; impact = 0.68), glycine, serine and threonine metabolism (FDR = 0.00032; impact = 0.55), and starch and sucrose metabolism (FDR = 0.00084; impact = 0.51). Similarly, citrate cycle (TCA cycle) (FDR = 0.00013; impact = 0.19) and pyruvate metabolism (FDR = 0.00036; impact = 0.30) were significantly affected.

**Fig. 5:**
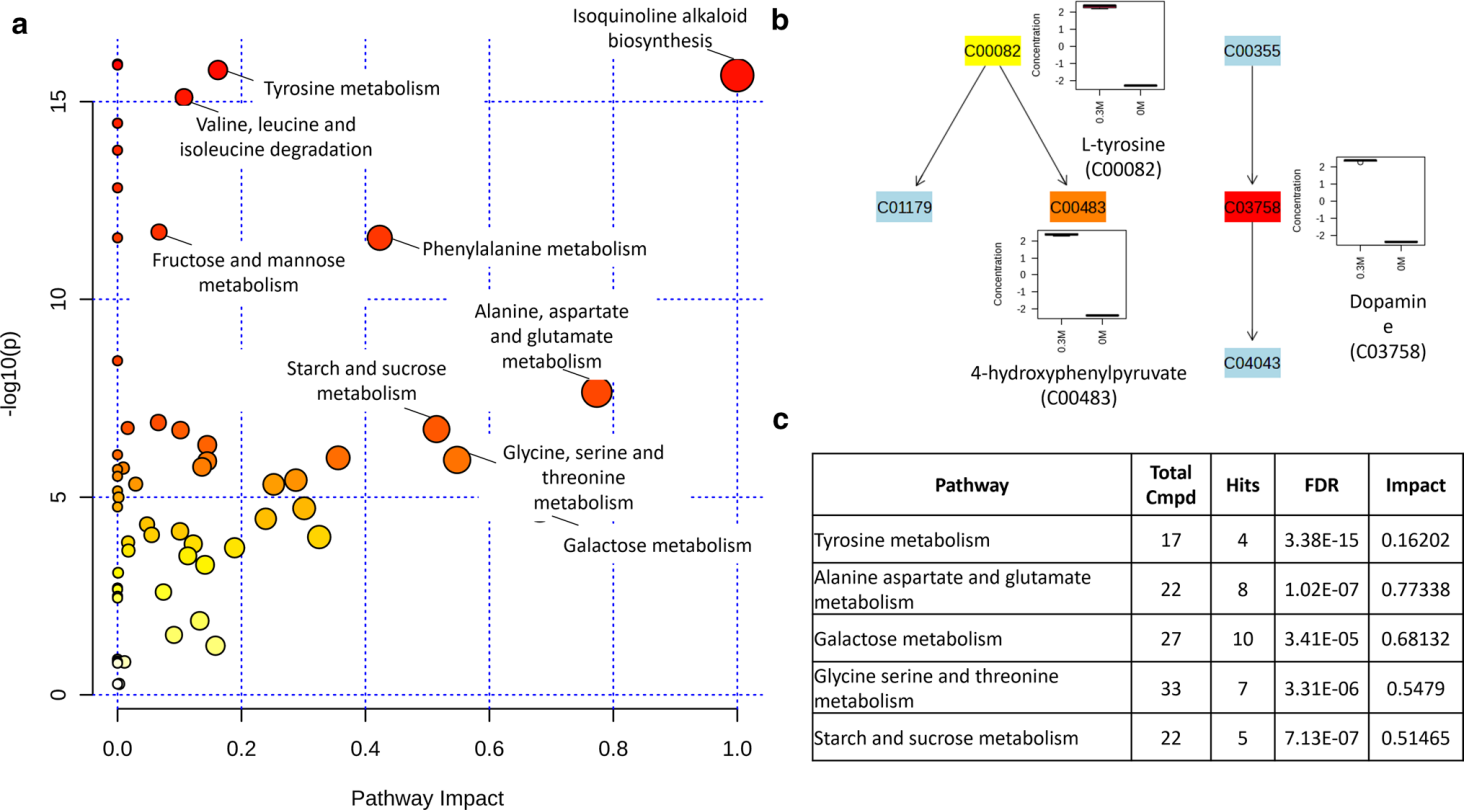
Pathway enrichment analysis of salt-induced metabolic changes between control (0 M) and the highest salinity treatment (0.3 M NaCl). **A** Bubble plot of pathway analysis results showing the most significantly enriched metabolic pathways. The *x*-axis represents pathway impact (topological analysis), while the *y*-axis represents pathway enrichment (–log₁₀(*p* value)). Bubble size reflects the number of matched compounds, and color intensity indicates statistical significance (FDR). **B** Isoquinoline alkaloid biosynthesis in cluster. Light blue compounds were not detected in our samples but are included as reference metabolites for the enrichment background; in contrast, compounds shaded from yellow to red represent metabolites putatively detected in our samples, with color intensity indicating the degree of statistical significance. **C** Table showing impact scores for the top five most affected pathways

Finally, the metabolic pathway analysis comparing control (0 M) and the highest salinity treatment (0.3 M NaCl) revealed a total of 53 significant pathways (Suppl. Table S14), many of which are functionally related to amino acid metabolism, sugar processing, and stress-related specialized metabolism. Among the top-scoring pathways (Fig. 5A–C), isoquinoline alkaloid biosynthesis showed the strongest enrichment (FDR = 3.44 × 10⁻^15^), with a pathway impact of 1.0, involving key precursors, such as l-tyrosine (C00082) and its conversion to 4-hydroxyphenylpyruvate (C00483) and tyramine (C01179) (Fig. 5B). Similarly, the alanine, aspartate, and glutamate metabolism pathway exhibited a high number of matched metabolites (8 hits out of 22 compounds), with strong significance (FDR = 1.02 × 10⁻⁷) and a substantial impact score (0.77). Additional enhanced pathways that are important in energy balance and osmoprotection under stress included the metabolism of galactose, glycine, serine, and threonine, as well as starch and sucrose. Interestingly, the biosynthesis pathways of amino acids, including valine, leucine, and isoleucine, as well as phenylalanine, tyrosine, and tryptophan, also considerably impacted, possibly indicating alterations in nitrogen allocation and phenylpropanoid-related metabolism.

## Discussion

### External stability vs. internal cost

Despite exposure to NaCl concentrations up to 0.3 M, *L. irtaense* did not show many significant reductions in growth traits, suggesting an apparent stability of vegetative performance. However, the observed downward trends in the mean values of biomass parameters are consistent with potential physiological costs under salt stress. Therefore, this apparent stability should be interpreted with caution. Almost all growth parameters, including root lengths, were found to be stable even at high salinity levels, while only minor reductions in root and leaf fresh weights were recorded. In addition, leaf water content remained unchanged, indicating the effective maintenance of turgor pressure by the plants. Conversely, the number of leaves decreased from 0.05 M NaCl onwards, with similar reductions across all applied NaCl concentrations. This reduction in leaf number observed in other *Limonium* species (Mir et al. 2022) can be considered a strategy to allocate resources to developing stress tolerance, as it stabilizes at higher salinity levels.

The results of the present work also support a previous study concerning the response of *L. irtaense* seedlings to high salinity, in which moderate reductions in growth were recorded, depending on the level of salinity. In addition, seedlings derived from seeds germinated in the presence of relatively low salt concentrations (0.05–0.1 M NaCl) showed better growth compared to those obtained from seeds germinated in water, indicating that mild and moderate stress can enhance tolerance in the early stages of development (Mircea et al. 2026).

The response of *L. irtaense* to salinity is similar to that of other *Limonium* species, which can vary from one taxon to another. For example, moderate reductions in biomass were recorded for *L. angustebracteatum* grown in high salinity, up to 0.8 M NaCl (Mir et al. 2022), and *L. albuferae* thrives in 0.2–0.4 M NaCl (González-Orenga et al. 2019a). In contrast, *L. dufourii* and other *Limonium* species are more sensitive to salt (González-Orenga et al. 2021).

### Photosynthetic stability and osmotic–antioxidant balance

Biochemical and metabolic analyses of *L. irtaense* under salt stress may reflect a relatively high degree of salt tolerance, as evidenced by the minimal degradation of photosynthetic pigments across the tested salinity gradient. This stability suggests that photosynthetic efficiency remains largely unaffected under moderate salinity conditions, which aligns with the observed maintenance of growth as reported in other species of this genus (Al Hassan et al. 2017; González-Orenga et al. 2019b; Mir et al. 2023). In juvenile *L. irtaense* plants, exposure to salt also triggered an increase in photosynthetic pigments (Mircea et al. 2026), similar to the response observed in two-week-old *L. sinuatum* seedlings exposed to 0.25 M NaCl (Xu et al. 2021). The most prominent biochemical adjustment was the strong dose-dependent accumulation of proline, which increased almost 30-fold at 0.3 M NaCl, reaching absolute concentrations high enough to be relevant for osmotic adjustment. Proline, a well-known osmoprotectant, plays a key role in mitigating stress by stabilizing proteins and membranes, scavenging reactive oxygen species (ROS), and as a signaling molecule, apart from contributing to maintain osmotic balance (Szabados and Savouré 2010; Alvarez et al. 2022). Its marked accumulation under high salinity reinforces its central role in stress tolerance, consistent with responses observed in other *Limonium* species (Tabot and Adams 2014; González-Orenga et al. 2020; Jang et al. 2023). Flavonoid content also rose progressively with salinity, supporting their protective role as secondary antioxidants. In contrast, GB levels showed a modest increase at 0.3 M NaCl but were too low to play any significant function in osmotic adjustment in salt-treated L. *irtaense* plants, as previously observed in seedlings (Mircea et al. 2026). This contrasts with other halophytes where GB plays a central role in osmoprotection (Etesami and Beattie 2018).

### Metabolic trade-offs under salinity

Carbohydrate metabolism in *L. irtaense* showed a clear stress-dependent adjustment. Total soluble sugars exhibited a biphasic response: they accumulated at low salinity but decreased significantly at high salinity, suggesting that in this species they are not consistently accumulated as osmolytes but, instead consumed or redirected under severe stress. Similar variability in soluble sugar responses has been reported in other Mediterranean *Limonium* species (Gil et al. 2013; González-Orenga et al. 2019b), indicating that their role in stress tolerance is not universal but may be species-specific or depend on the stress intensity.

Many salt-stressed plants accumulate sugars and sugar alcohols to concentrations high enough to have osmotic effects (Gil et al. 2013). Even among closely related taxa, such as those in the genus *Limonium*, where different metabolite accumulation patterns have been documented, species-specific differences are frequent (Gagneul et al. 2007; Liu and Grieve 2009; González-Orenga et al. 2019b). In *L. irtaense*, metabolomic analysis revealed a general decline in carbohydrate levels with increasing salinity, potentially due to their utilization as energy sources to support stress responses or conversion into osmoprotectants (Keunen et al. 2013). Some carbohydrates and sugar derivatives were consistently down-accumulated across salt treatments. Notably, lactobionic acid, a polyol acid with antioxidant properties not commonly associated with plant metabolism (Goderska 2019), was significantly abundant in control plants, suggesting its accumulation was linked to unstressed physiological states. Similar declines were observed for galactose, psicose, and dehydroabietic acid. Galactose, involved in cell wall biosynthesis and osmotic regulation (Pérez-Almeida and Carpita 2006; Hu et al. 2020), may be diverted into structural or protective pathways under stress. Although galactose accumulation in response to salt has been reported in other studies (Joshi et al. 2005), its reduction here suggests species-specific metabolic adaptations. Psicose, an uncommon sugar associated with disease resistance in rice (Kano et al. 2011) and accumulated under salt and drought stress (Yadav et al. 2021), also declined here, suggesting a different prioritization of metabolic pathways. The role of dehydroabietic acid remains unclear though it may be associated with lipid signaling or membrane remodeling. Further characterization is needed to confirm these possibilities.

In contrast, sugar alcohols, such as xylitol and meso-erythritol, accumulated strongly with increasing NaCl concentrations. These polyols are well known to stabilize membranes and proteins, contribute to osmotic balance, and mitigate oxidative stress (Pamuru et al. 2021; Chen et al. 2025). The contrasting behavior of sugars and polyols reflects a metabolic trade-off, in which carbon is shifted from primary energy pools toward compounds with stronger protective functions. An additional adjustment involved octacosane upregulation under salt stress, a long-chain alkane associated with cuticular wax deposition, previously reported in *Limonium* species (Shepherd and Griffiths 2006).

Oxidative stress markers also suggest a significant impact of salinity on cellular integrity. The linear increase in MDA concentration with escalating salinity suggests enhanced lipid peroxidation, which is characteristic of oxidative stress (Ozgur et al. 2013). MDA levels increased in response to increasing salinity in several halophytes, including *Cakile maritima* (Ksouri et al. 2007), *Sesuvium portulacastrum* (Lokhande et al. 2011), *Gypsophila oblanceolata* (Sekmen et al. 2012), or *L. stocksii* (Hameed et al. 2015). Nonetheless, the relatively stable levels of hydrogen peroxide (H_2_O_2_) indicate that antioxidant systems are successfully reducing oxidative damage, especially in the presence of moderate salinity, which is in line with observations made in other halophytes (Aghaleh et al. 2011; Nisar et al. 2021). This suggests that, to counteract oxidative stress under moderate salinity, *L. irtaense* has developed strong antioxidant defenses, which may be less effective under higher salinity conditions.

### Selective ion regulation

Ion accumulation patterns provided additional insights into the plant’s salt tolerance mechanisms. As expected, sodium (Na⁺) and chloride (Cl⁻) concentrations increased strongly with salinity, particularly in leaves, reflecting active translocation from roots to aerial tissues. This strategy, common in halophytes, facilitates toxic ion sequestration in vacuoles and—in the case of recretohalophytes such as most *Limonium* species—ion excretion through salt glands, while preventing excessive accumulation in roots (Mi et al. 2021). Salinity-induced potassium (K⁺) depletion is a common stress response due to competition between Na⁺ and K⁺ for transport sites (Shabala 2003). Salt-tolerant species typically sustain higher K⁺ concentrations to preserve cellular function under salt stress (Zhu 2001). In *L. irtaense*, K⁺ levels in leaves remained relatively high compared to roots across all treatments, supporting this adaptive strategy and aligning with observations in many halophytes, such as *Cakile maritima* (Ellouzi et al. 2011) and several *Limonium* species (González-Orenga et al. 2020, 2021). Interestingly, while root K⁺ concentrations were stable under moderate salinity, a significant increase in root K⁺ content was observed at 0.3 M NaCl, suggesting an active salt tolerance mechanism. This response likely represents an adaptive strategy at high salinity, where *L. irtaense* retains K⁺ in the roots to maintain a more negative water potential, which facilitates continued water uptake despite osmotic pressure. Although Na⁺ typically competes with K⁺ for uptake, leading to a reduction in cellular K⁺ levels under increasing salinity, many *Limonium* species actively transport K⁺ to the leaves, maintaining or even enhancing leaf K⁺ levels under saline stress (Al Hassan et al. 2017). Our previous study on *L. irtaense* seedlings further supports this notion, showing a significant increase in K⁺ in both roots and leaves under salt stress, highlighting K⁺ retention as a key mechanism for tolerance (Mircea et al. 2026). The observed increase in root K⁺ at 0.3 M NaCl suggests that *L. irtaense* employs a strategy of active K⁺ accumulation in the roots, potentially facilitated by specialized K⁺ transporters. This allows the plant to maintain osmotic balance under extreme salinity, a critical survival trait in saline environments. The importance of K⁺ retention in roots as a mechanism for salt tolerance was highlighted by Shabala and Cuin (2008), which demonstrated a strong positive correlation between a plant’s ability to retain K⁺ in the roots and its overall salinity tolerance. This mechanism is not only crucial for halophytes but also for glycophytes, where K⁺ retention has been linked to improved osmotic homeostasis and enhanced metabolic function in root cells under saline stress (Wu et al. 2015).

In contrast, the decline in leaf Ca^2^⁺ content at higher salinity may signal disruptions in membrane stability and calcium-dependent signaling pathways, contributing to growth inhibition. However, overall Ca^2^⁺ uptake by roots appeared relatively unaffected across salinity treatments, suggesting that root-level calcium absorption was less sensitive to salt stress.

### Integration of nitrogen metabolism and tyrosine-derived pathways

At the highest salinity level tested (0.3 M of NaCl), the putatively annotated metabolomic profile of *L. irtaense* revealed not only the accumulation of classical osmoprotectants but also a marked increase in several nitrogen-containing metabolites, including glutamine, glutamate, and asparagine. These amino acids are closely linked to primary nitrogen assimilation pathways (Coruzzi 2003), suggesting a shift toward supporting nitrogen assimilation and metabolism under severe salt stress. The increased abundance of these compounds may indicate the activation of compensatory mechanisms involved in nitrogen assimilation and the maintenance of cellular homeostasis even under ionic disequilibrium.

A notable feature of *L. irtaense* under salt stress is the consistent increase in catecholamine-related compounds, especially dopamine; similar results have been documented in the halophytic grass *Puccinellia nuttalliana* under salt stress (Vaziriyeganeh et al. 2021), and in *Citrullus colocynthis* and *Haloxylon articulatum* (Jdey et al. 2017). The consistent increase of both dopamine and normetanephrine at different salinity levels in *L. irtaense* may therefore represent a distinctive metabolic signature, potentially extending current evidence for catecholamine involvement in halophyte salt tolerance beyond what has been observed in other species.

Although such metabolites are rarely detected in halophytes, they may play an important physiological role in the response to salt treatments. Dopamine pretreatment has been demonstrated to reduce salt stress in plants (Li et al. 2015), and it has been linked to ROS scavenging and regulatory functions under both biotic and abiotic stress (Gill and Tuteja 2010; Liu et al. 2020; Ahammed and Li 2023; Saharan et al. 2024). In addition to its antioxidant role, dopamine appears to participate in ionic regulation under salt stress by increasing K⁺/Na⁺ and Ca^2^⁺/Na⁺ ratios, suggesting a role in maintaining ion balance and membrane stability (Yildirim et al. 2024). In *P. nuttalliana*, dopamine was proposed to improve antioxidant capacity by scavenging ROS, regulating SOS1 expression, and activating Ca^2^⁺ signaling pathways (Vaziriyeganeh et al. 2021). Therefore, the strong increase in dopamine observed in *L. irtaense* suggests a potential contribution to stress tolerance that aligns with mechanisms observed in both halophytic and glycophytic species.

Beyond these protective functions, dopamine may also influence nitrogen metabolism. Previous studies in both glycophytes and halophytes have reported that high salinity can impair nitrate uptake through interactions with nitrate transporters (Rubinigg et al. 2003, 2005; Liu et al. 2024). However, the increased abundance of nitrogen-related metabolites in *L. irtaense* suggests the activation of compensatory mechanisms that support nitrogen assimilation even under ion imbalance. Dopamine has been associated with the upregulation of nitrate transporter genes and promotion of stress tolerance (Li et al. 2015; Liu et al. 2020; Ahammed and Li 2023), which may partially explain the observed increase in amino acid biosynthesis pathways at 0.3 M NaCl.

Tyramine, produced by tyrosine decarboxylation, was also elevated under salt stress conditions. It is linked to cell wall modification and ion transport regulation, likely through its involvement in the biosynthesis of hydroxycinnamic acid amides and other phenolic compounds bound to the cell wall (Macoy et al. 2015). Its accumulation under high salinity conditions suggests that *L. irtaense* may strengthen structural barriers and adjust cation homeostasis as part of its defense strategy. The co-occurrence of dopamine, normetanephrine, and tyramine indicates that *L. irtaense* may activate a tyrosine-derived metabolic module that complements classical tolerance mechanisms. This module integrates antioxidant defense, ion regulation, and structural protection, adding an additional layer of resilience. Since catecholamines have rarely been identified in *Limonium* species, their consistent induction in *L. irtaense* may indicate a relevant and species-specific feature, with potential value as a candidate biochemical marker of salinity tolerance in narrowly distributed halophytes. Nevertheless, the identification of catecholamine and related compounds should be considered as putative as it relied only on matching spectra from a database, and should be confirmed to support their use as salt stress biomarkers.

### Dose-dependent metabolic reprogramming

Pathway enrichment analysis indicated that *L. irtaense* undergoes a progressive increase in the number and complexity of affected pathways: from only two significantly enriched pathways at 0.05 M to 53 at 0.3 M NaCl, suggesting a gradual yet extensive metabolic reorganization as stress intensified. At low salinity, early activation of glutathione metabolism and betalain biosynthesis was observed. These pathways are associated with redox homeostasis and early stress signaling (Sabir et al. 2025), suggesting that *L. irtaense* rapidly engages detoxification systems upon low salt exposure. Moderate salinity (0.1–0.2 M) was associated with changes in carbohydrate metabolism (e.g., galactose, starch, sucrose, and glycerolipids), energy pathways (TCA cycle, glycolysis), and amino acid biosynthesis (e.g., glutamate, serine/threonine). Together, these shifts suggest a metabolic transition that may reallocate carbon and nitrogen resources from growth-related functions toward stress mitigation. At the highest salinity tested (0.3 M NaCl), *L. irtaense* displayed a pronounced enrichment of specialized metabolic pathways, particularly isoquinoline alkaloid biosynthesis derived from tyrosine intermediates, such as 4-hydroxyphenylpyruvate and tyramine. This pattern suggests a shift toward the synthesis of antioxidant and structural defense compounds, consistent with responses observed in other halophytes such as *Kosteletzkya pentacarpos* (Mircea et al. 2025).

## Conclusion

This study provides the first integrative physiological, biochemical, and metabolic characterization of salt tolerance in *Limonium irtaense*. The species maintains an apparently stable growth phenotype under salinity, despite underlying physiological stress signals. This tolerance is sustained through coordinated biochemical and metabolic adjustments and selective ion transport regulation. Metabolomic analysis revealed a progressive reprogramming of primary and specialized metabolism, with early osmotic and antioxidant responses followed by activation of tyrosine-derived and amino acid-related pathways, potentially associated with structural and redox defense mechanisms. These results suggest that salt tolerance in *L. irtaense* is not the result of insensitivity, but of a flexible metabolic reallocation that preserves cellular functions under ionic and oxidative stress. The identified metabolites, proline, and flavonoids, together with the putatively annotated polyols, and catecholamines, represent candidate biochemical markers for monitoring population status and guiding conservation efforts. More broadly, *L. irtaense* offers a valuable model for understanding how endemic halophytes balance resilience and metabolic cost in saline environments.

## Supplementary Information

Below is the link to the electronic supplementary material.

Supplementary file1 (XLSX 135 KB)

## Data Availability

Data are available at Mendeley Data: https://data.mendeley.com/datasets/y3r7rrn6zf/2

## Abbreviations

GB: Glycine betaine
MDA: Malondialdehyde
PCA: Principal component analysis
Pro: Proline
TF: Total flavonoids
TPC: Total phenolic compounds
TSS: Total soluble sugars
WC: Water content
